# Supplementary material for: AI for radiographic COVID-19 detection selects shortcuts over signal
Source: medRxiv. 2020 Oct 7:2020.09.13.20193565. Originally published 2020 Sep 14. Preprint. [Version 2] doi: 10.1101/2020.09.13.20193565 (PMC7523163; doi:10.1101/2020.09.13.20193565)
Supplement: 1 [file NIHPP2020.09.13.20193565-supplement-1.pdf]

medRxiv preprint doi: https://doi.org/10.1101/2020.09.13.20193565.this version posted October 8, 2020. The copyright holder for this preprint (which was not certified by peer review) is the author/funder, who has granted medRxiv a license to display the preprint in perpetuity.
It is made available under a CC-BY 4.0 International license .

# Supplementary Information

## Supplementary Note

While saliency maps are widely used to interpret image-based artificial intelligence systems [32, 33, 46], the reliability of these approaches has been disputed by contemporary work, which observes that saliency maps explaining medical imaging classifiers fail to localize medically relevant pathology [47]. However, this prior work did not disentangle whether (i) the saliency maps fail to identify the features that are important for the classification models, or (ii) the saliency maps faithfully identify the features that are important for the classification models, but the models do not depend on medically relevant pathology. We hypothesised the latter, that attribution maps fail to localize relevant pathology because the models they explain do not rely on relevant pathology [48].

To validate that the pixels selected by our saliency maps are truly important for the models they explain, we chose 100 images that our model predicted are COVID-19 negative, then masked and mean-imputed a subset of pixels. If we selected these pixels at random, we would expect the models output to regress to the mean output (become more positive) since the negative images become more like the mean image (which is predicted to be more positive than the COVID-19 negative images). If the pixels identified by Expected Gradients are important for the model's prediction, we would anticipate that masking these pixels should make the model's output *more positive* than masking randomly selected pixels. When we mask the top 10% of pixels identified by EG as contributing to the negative prediction of the model, we see that the model's output is shifted to be significantly more negative than when we mask pixels selected at random (Supplementary Fig. 4).

## Supplementary Figures

| | **Dataset I** | | | **Dataset II** | | |
|---|---|---|---|---|---|---|
| | Combined | CXR14 | Cohen et al. | Combined | PadChest | BIMCV-COVID |
| CXR #s | 112,528 | 112,120 | 408 | 97,866 | 96,270 | 1,596 |
| Patients, #s | 31,067 | 30,805 | 262 | 64,954 | 63,939 | 1,015 |
| Age, mean (std) | 46.9 (16.8) | 46.9 (16.8) | 57.0 (16.4) | 65.4 (20.1) | 65.5 (20.1) | 61.2 (16.0) |
| Sex, N women (%) | 48,926 (43.5) | 48,780 (43.5) | 146 (35.8) | 49,700 (50.8) | 49,010 (50.9) | 690 (43.2) |
| AP Images (%) | 44,916 (39.9) | 44,810 (40.0) | 106 (26.0) | 5,485 (5.6) | 4,557 (4.7) | 928 (58.1) |
| COVID + (%) | 312 (0.2) | 0 (0.0) | 312 (76.5) | 1,596 (1.6) | 0 (0.0) | 1,596 (100.0) |
| Non-COVID Pneumonia (%) | 1,494 (1.3) | 1,413 (1.3) | 81 (19.9) | 4,145 (4.2) | 4,145 (4.3) | 0 (0.0) |

**Supplementary Table 1** | Summary characteristics of our two main datasets (multi-source and single-source), as well as the summary characteristics of the data sources that are combined to yield these datasets.

medRxiv preprint doi: https://doi.org/10.1101/2020.09.13.20193565.this version posted October 8, 2020. The copyright holder for this preprint (which was not certified by peer review) is the author/funder, who has granted medRxiv a license to display the preprint in perpetuity.
It is made available under a CC-BY 4.0 International license .

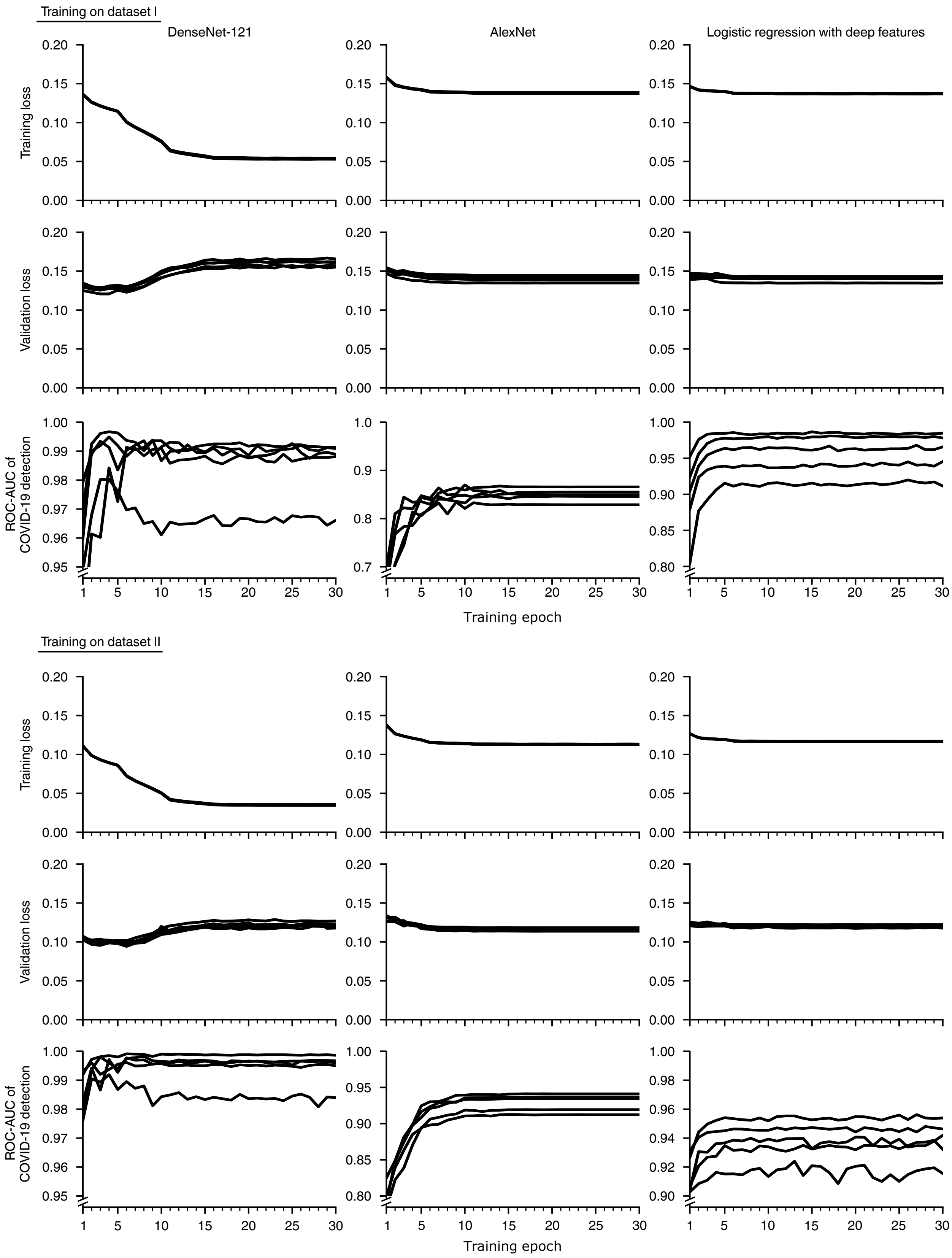


**Supplementary Fig. 1** | (Caption next page.)

medRxiv preprint doi: https://doi.org/10.1101/2020.09.13.20193565.this version posted October 8, 2020. The copyright holder for this preprint (which was not certified by peer review) is the author/funder, who has granted medRxiv a license to display the preprint in perpetuity.
It is made available under a CC-BY 4.0 International license .

**Supplementary Fig. 1 | (Previous page.) Evolution of metrics that monitor the artificial neural network training process.** Training curves are shown for each of 5 random train/validation/test splits of the datasets. During the training procedure, the model is progressively optimized to decrease the training loss, for which we chose the *binary cross entropy.* The validation loss monitors the same metric on a subset of the training radiographs that is held-out from the optimization process (and that is also entirely separate from testing data). Increases in the validation loss may indicate that the model has *overfit* the training data, *i.e.*, the model has memorized the training data rather than learning general principles that apply to new radiographs, such as those in the validation set. To prevent overfitting, we save models when they achieve a maximum in the area under the receiver operating characteristic curve (ROC-AUC) for COVID-19 classification in the held-out validation set, and we use these models for all subsequent analysis. All models were trained for a total of 30 epochs, which was sufficient to attain a maximum in the ROC-AUC of COVID-19 classification. Note that to permit visualization of the maximum in the ROC-AUC of COVID-19 detection, the plots that visualize this quantity feature variable y-axis scales.

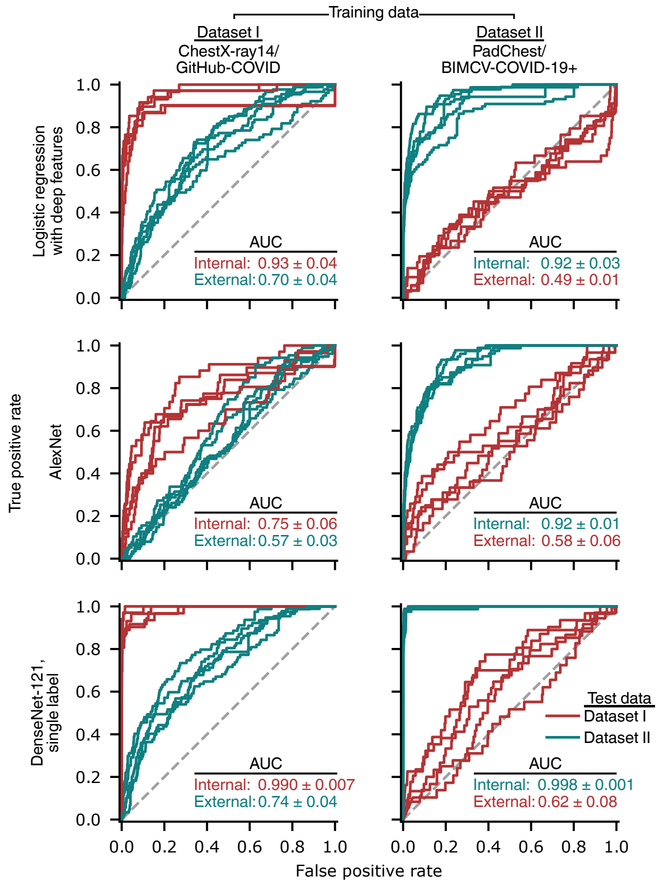


**Supplementary Fig. 2 | Generalization performance of alternative models, as measured by receiver-operating characteristic (ROC) curves.** The first two rows correspond to models in which the capacity to overfit, which has been implicated in learning of spurious associations [29], has been reduced. The logistic regression with deep features comprises a neural network with the DenseNet-121 architecture that was trained on the ImageNet dataset to derive a set of of 1024 general image features, *i.e.* those output by the penultimate layer of the network, which were used as inputs for a logistic regression; the weights of the neural network were held fixed during training of the logistic regression. The AlexNet models follow the original AlexNet model architecture [15] but with the final 1000-class classification head replaced by a 15-class classification head, corresponding to the 14 ChestX-ray14 labels plus an additional label for COVID-19. The final row represents models with an identical architecture and training scheme to those in the main text, except with only a single output corresponding to presence/absence of COVID-19. Red and teal numbers indicate area under the ROC curves (AUC, mean ± standard deviation, $n$=5).

medRxiv preprint doi: https://doi.org/10.1101/2020.09.13.20193565.this version posted October 8, 2020. The copyright holder for this preprint
(which was not certified by peer review) is the author/funder, who has granted medRxiv a license to display the preprint in perpetuity.
It is made available under a CC-BY 4.0 International license .

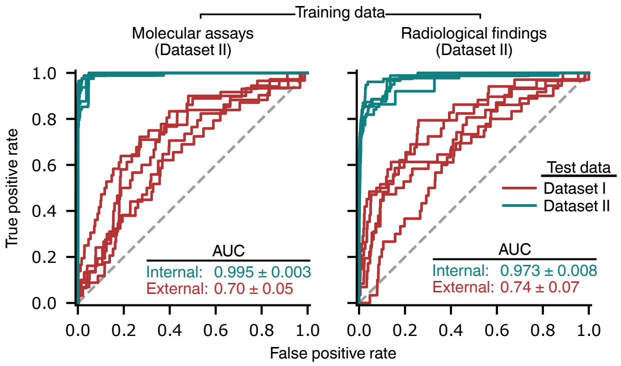


**Supplementary Fig. 3 | Evaluation of the impact on generalization performance of *concept shift*, a change in the classification task between the training and testing datasets.** In addition to the learning of spurious correlations that do not remain constant between datasets, generalization performance may also drop due to changes in non-spurious correlations between datasets, including a shift in how the labels are generated. In particular, the GitHub-COVID dataset [20], which consists largely of radiographs published in academic articles, may predominantly feature COVID-19+ images with radiological evidence of COVID-19, while COVID-19 labels for the BIMCV-COVID-19+ dataset [23] may be derived from molecular assays (left panel), including reverse-transcription polymerase chain reaction and serology, or from a radiologist's assessment for radiological evidence of COVID-19 (right panel) in addition to confirmation by molecular assays. Specifically, we defined "radiological evidence of COVID-19" as presence of *COVID-19* or *COVID-19 uncertain* in the radiologist-derived labels of BIMCV-COVID-19+. In the event that poor generalization performance is due to a shift from predicting presence of COVID-19, with or without radiological evidence, in the training data, to predicting radiological evidence of COVID-19 in the test data, generalization performance would be expected to increase substantially. Red and teal numbers indicate area under the ROC curves (AUC, mean ± standard deviation, $n$=5).

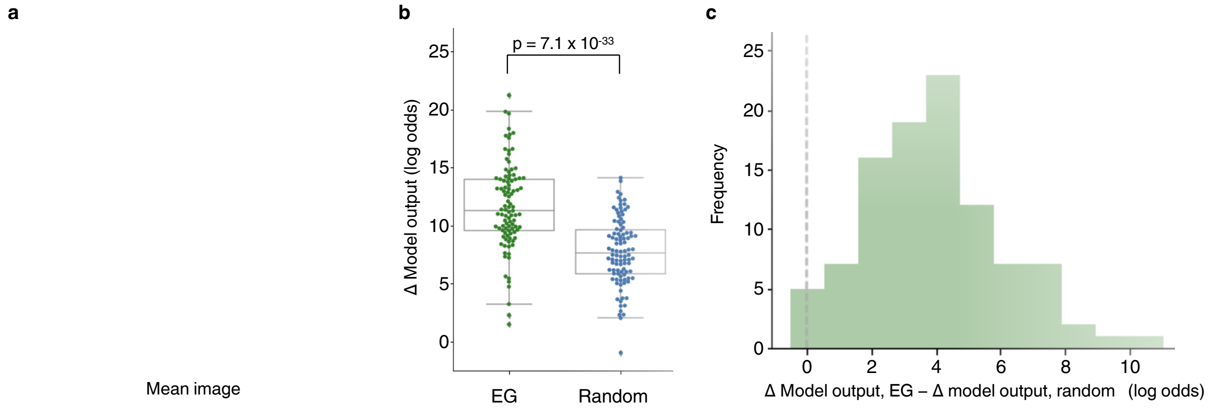


**Supplementary Fig. 4 | Ablation tests to assess the importance of pixels that are highlighted by saliency maps. a**, Average image of COVID-19+ radiographs from dataset I, from which pixels are drawn to "ablate", *i.e.*, hide, putatively important parts of individual radiographs in our experiment. **b**, Comparison of the change in an AI-based COVID-19 classification model's predictions when pixels are ablated based on their saliency map importance scores or by random. For a randomly chosen subset of radiographs, the 10% of pixels with the highest magnitude expected gradients (EG) scores were ablated by replacing those pixels with the corresponding pixels from the average COVID-19+ image, and as a control, an equivalent number of pixels were replaced at random. Note that in both cases, the model's predicted log odds that the radiograph represents a COVID-19+ patient is expected to increase, since pixels are replaced with pixels from the mean COVID-19+ image. The $p$-value is calculated by a two-sided Wilcoxon signed-rank test, $n$=100 ($W = 7.69$, $p = 1.48 \times 10^{-14}$. **c**, Pairwise comparison of the change in the model's predictions, to assess the superiority of EG relative to random choice at determining important pixels. Since the potential for ablation to change the model's prediction varies from image to image, overlap in the distributions of "EG" and "random" in **b** does *not* imply that for any given image random choice is superior to EG. If for any image a random choice of pixels were superior to EG at determining important pixels, we would expect to observe values less than zero in the histogram, which shows image-level, pairwise differences between EG and random choice.

medRxiv preprint doi: https://doi.org/10.1101/2020.09.13.20193565.this version posted October 8, 2020. The copyright holder for this preprint (which was not certified by peer review) is the author/funder, who has granted medRxiv a license to display the preprint in perpetuity.
It is made available under a CC-BY 4.0 International license .

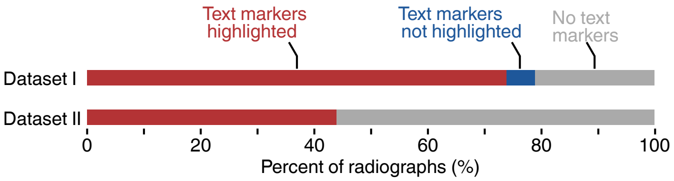


**Supplementary Fig. 5 | Analysis of the frequency at which saliency maps highlight laterality markers as important features.** To assess the frequency, a random sample of 100 radiographs and their corresponding saliency maps was chosen from each dataset, and each radiograph was manually categorized as (i) contains a laterality marker that is highlighted by the saliency map, (ii) contains a laterality marker that is not highlighted by the saliency map, or (iii) does not contain a laterality marker.

medRxiv preprint doi: https://doi.org/10.1101/2020.09.13.20193565.this version posted October 8, 2020. The copyright holder for this preprint
(which was not certified by peer review) is the author/funder, who has granted medRxiv a license to display the preprint in perpetuity.
It is made available under a CC-BY 4.0 International license .

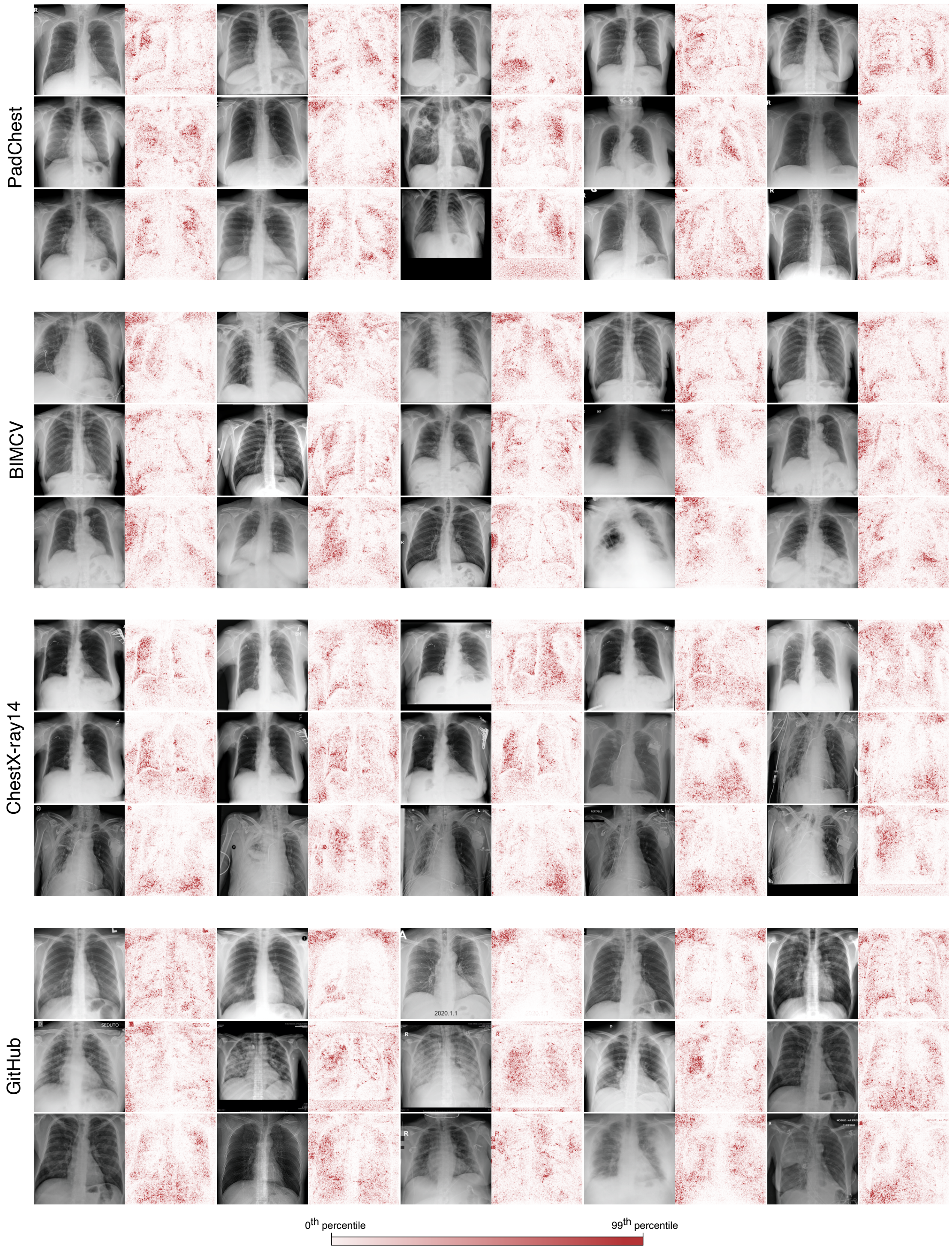


**Supplementary Fig. 6 | Saliency maps for 15 radiographs from each of the four data sources.** Across all four data sources, saliency maps highlight text tokens and laterality markers (e.g., the first radiograph-saliency map pair in the first row of the PadChest examples, the second-to-last and last radiograph-saliency map pairs in the third row of the PadChest examples, the first four radiograph-saliency map pairs in the second row of the BIMCV examples, all five radiograph-saliency map pairs in the third row of the ChestX-ray14 examples, and the first three radiograph-saliency map pairs in the first row of the GitHub examples).

medRxiv preprint doi: https://doi.org/10.1101/2020.09.13.20193565.this version posted October 8, 2020. The copyright holder for this preprint (which was not certified by peer review) is the author/funder, who has granted medRxiv a license to display the preprint in perpetuity.
It is made available under a CC-BY 4.0 International license .

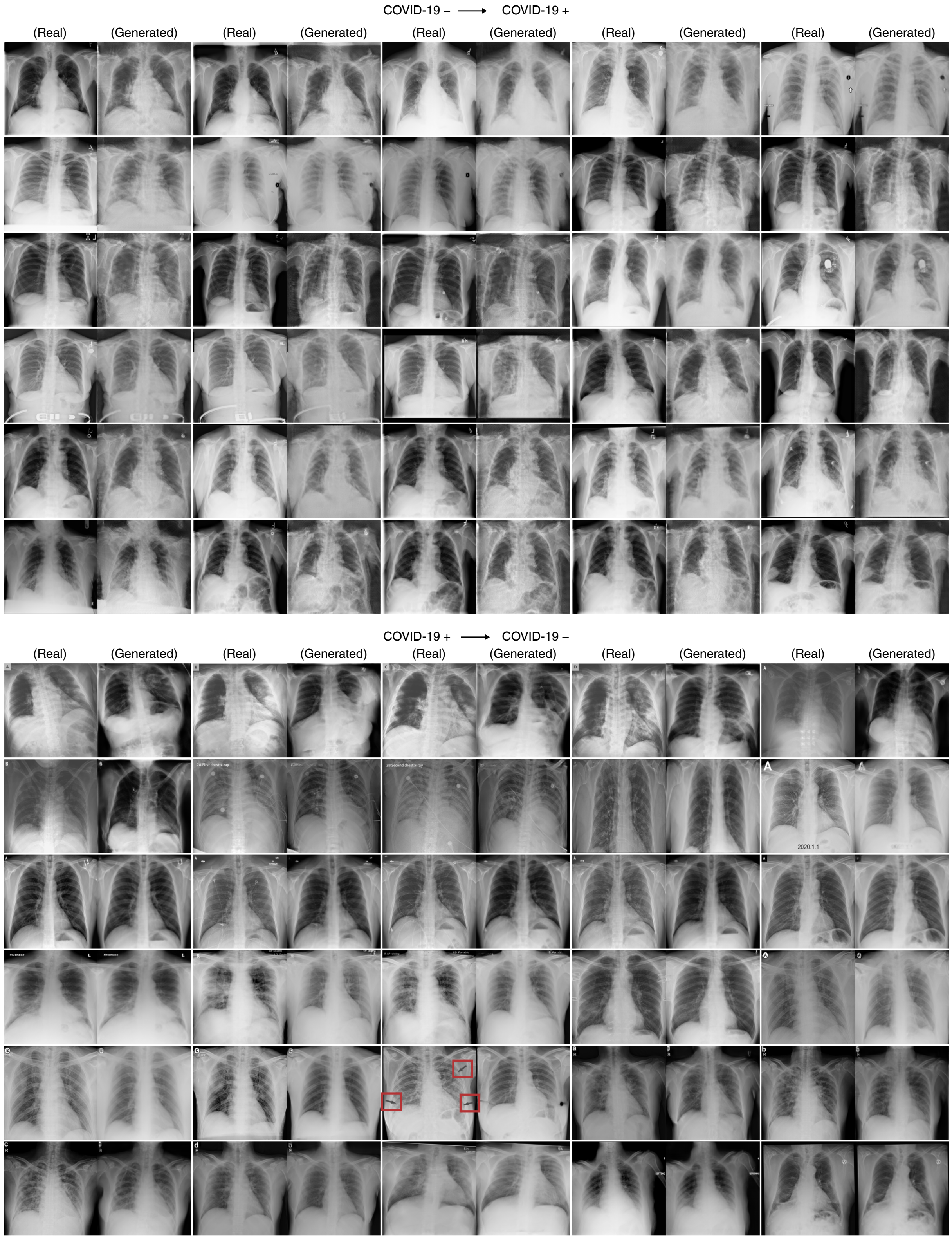


**Supplementary Fig. 7 | Examples images generated by a CycleGAN that was trained to alter COVID-19 negative images from the ChestX-ray14 dataset to appear like COVID-19 positive images from the GitHub-COVID dataset and vice versa.** Red boxes in lower panel mark annotations that were removed by the CycleGAN, as referenced in the main text. Images from the GitHub-COVID repository may contain annotations, as many were scraped from figures in academic publications.

medRxiv preprint doi: https://doi.org/10.1101/2020.09.13.20193565.this version posted October 8, 2020. The copyright holder for this preprint
(which was not certified by peer review) is the author/funder, who has granted medRxiv a license to display the preprint in perpetuity.
It is made available under a CC-BY 4.0 International license .

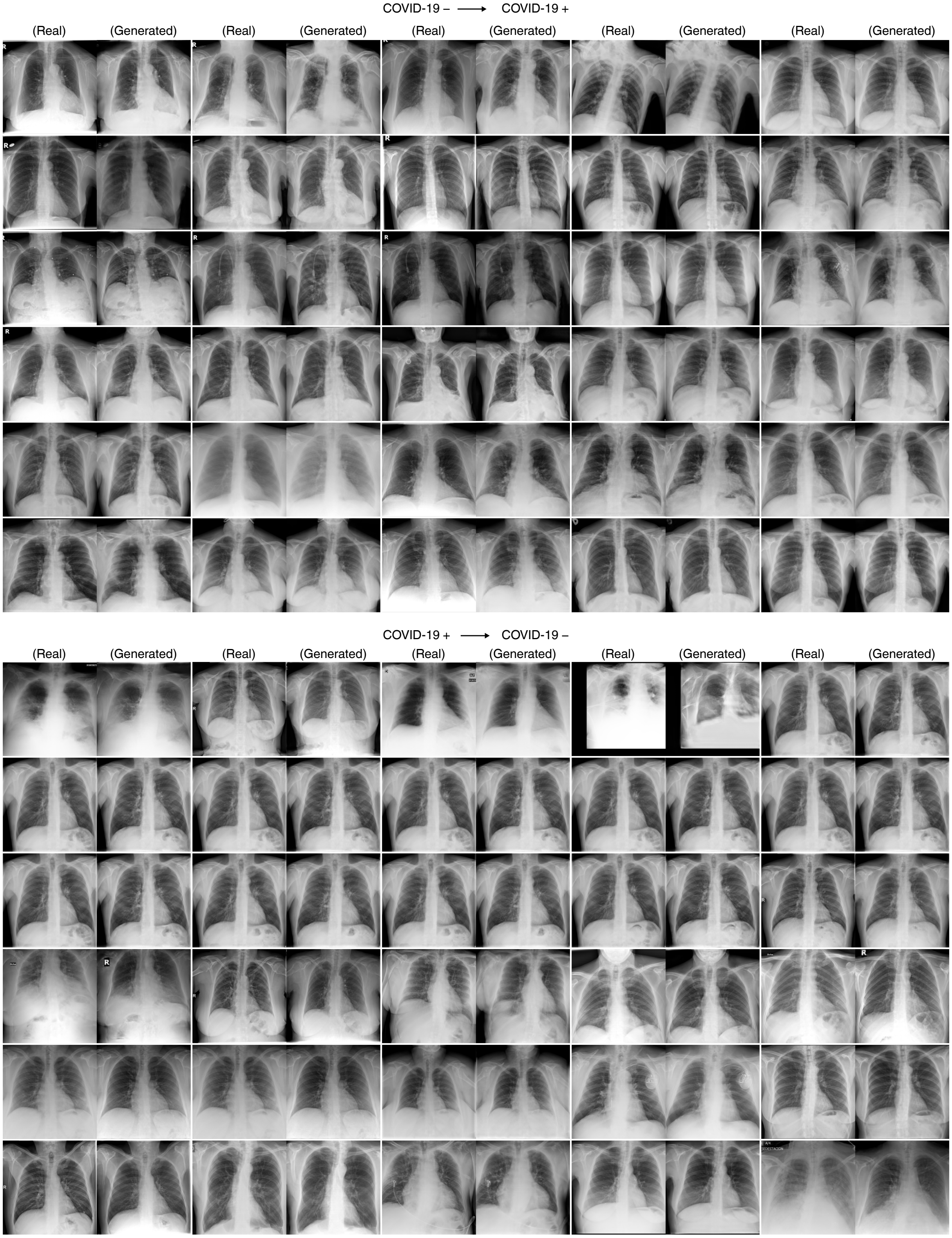


**Supplementary Fig. 8 | Examples images generated by a CycleGAN that was trained to alter COVID-19 negative images from the PadCheset dataset to appear like COVID-19 positive images from the BIMCV-COVID-19+ dataset and vice versa.**

medRxiv preprint doi: https://doi.org/10.1101/2020.09.13.20193565.this version posted October 8, 2020. The copyright holder for this preprint
(which was not certified by peer review) is the author/funder, who has granted medRxiv a license to display the preprint in perpetuity.
It is made available under a CC-BY 4.0 International license .

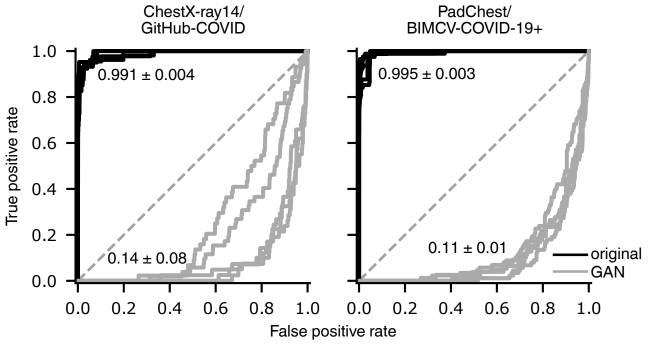


**Supplementary Fig. 9 | Evaluation of the extent to which features relied upon by the COVID-19 detection models are altered by the CycleGAN, as measured by the drop in classification performance following transformation by the CycleGAN.** A CycleGAN that more reliably alters images such that they appear to the classifier to be of the COVID-19 label opposite their original will achieve an area under the ROC curve (AUC) closer to zero. Inset values indicate AUC (mean ± standard deviation, $n$=5).

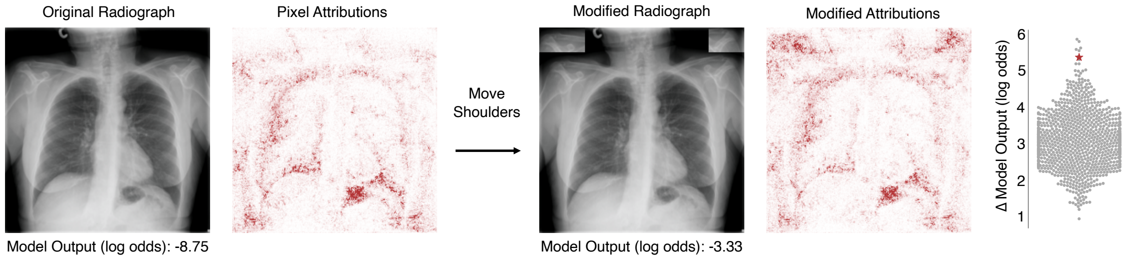


**Supplementary Fig. 10 | Additional assessment of the importance of shoulder positioning to an AI model for radiographic COVID-19 detection.** The procedure to generate Figure 2d was replicated with a new radiograph; *i.e.*, a patch of the radiograph containing the patient's clavicles was copied to the top corners of the image, and the increase in the model's predicted log odds of COVID-19 was compared to that produced by copying random image patches of the same size ($\Delta = 5.42$, empircal $p$-value $= 7 \times 10^{-3}$ based on Monte Carlo substitution of random image patches, $n$=1000) (see Methods Section 2.5).

medRxiv preprint doi: https://doi.org/10.1101/2020.09.13.20193565.this version posted October 8, 2020. The copyright holder for this preprint (which was not certified by peer review) is the author/funder, who has granted medRxiv a license to display the preprint in perpetuity.
It is made available under a CC-BY 4.0 International license .

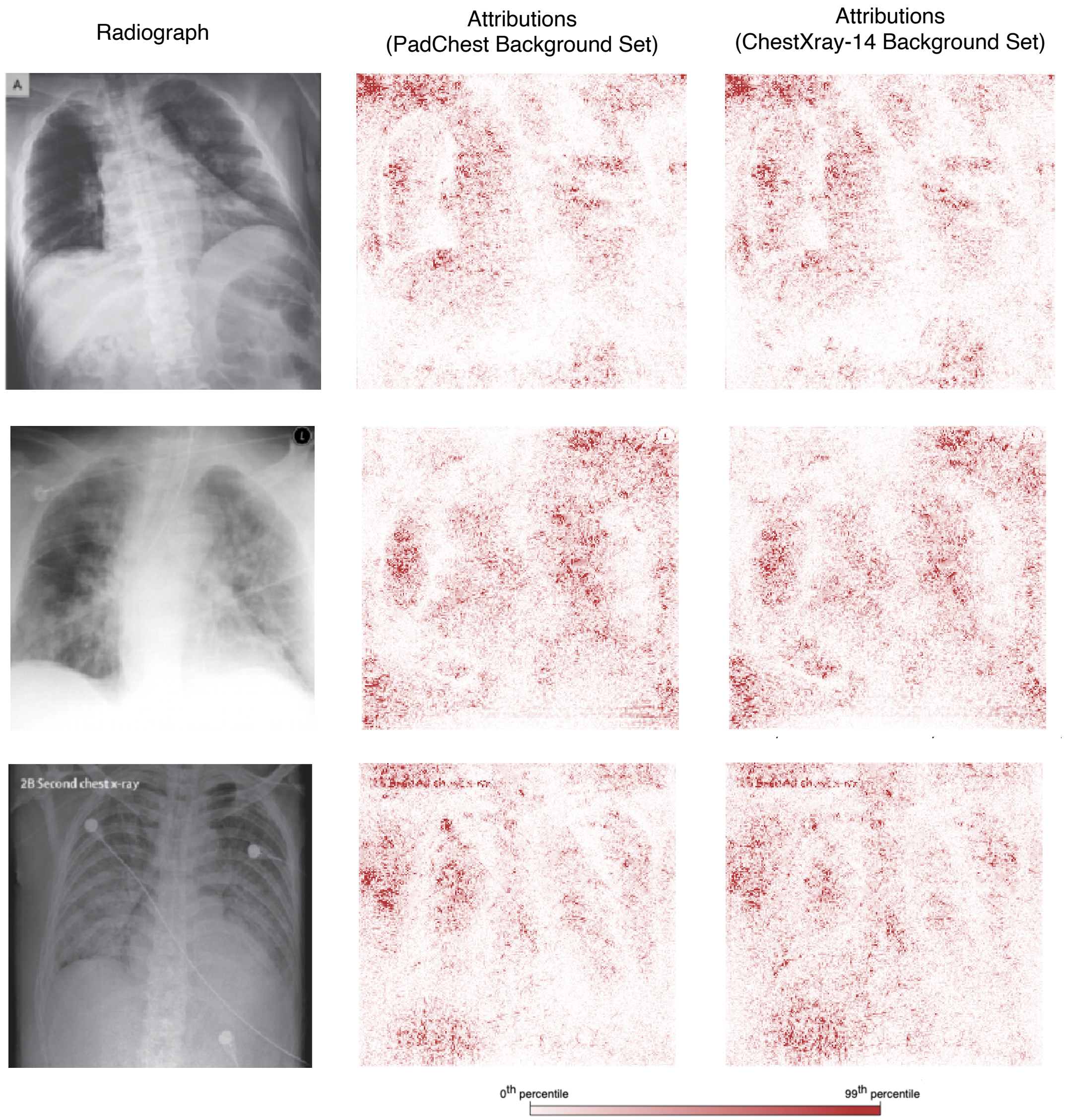


**Supplementary Fig. 11 | Comparison of expected gradients saliency maps generated from varied reference distributions, which provide the baseline radiographs from which the expected gradients algorithm integrates.**

medRxiv preprint doi: https://doi.org/10.1101/2020.09.13.20193565.this version posted October 8, 2020. The copyright holder for this preprint
(which was not certified by peer review) is the author/funder, who has granted medRxiv a license to display the preprint in perpetuity.
It is made available under a CC-BY 4.0 International license .

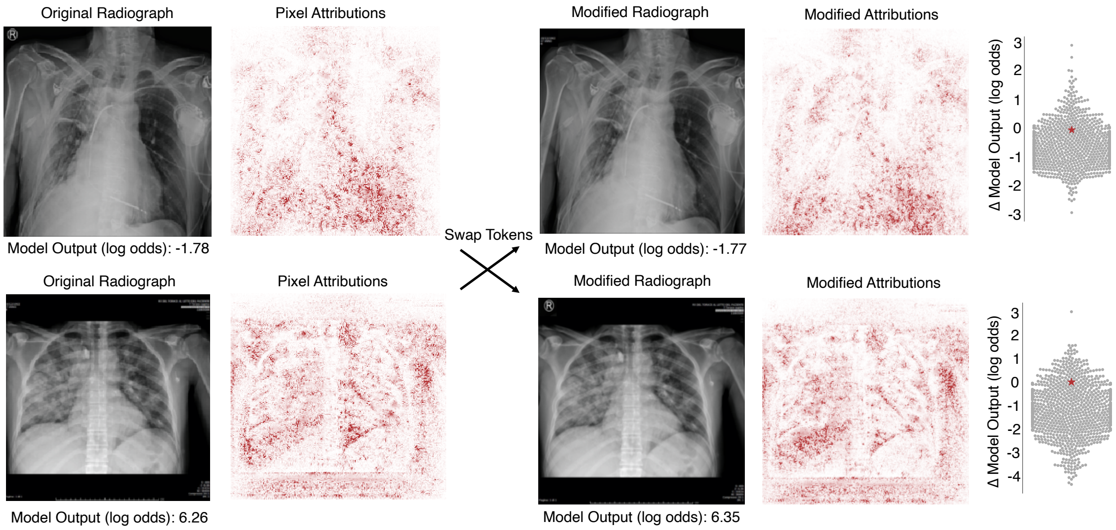


**Supplementary Fig. 12 | Evaluation of the impact on model output of text tokens that were not highlighted as important via saliency maps.** Following the procedure of Fig. 2c, text tokens of COVID-19 negative (upper row) and COVID-19 positive radiographs (lower row) were swapped, and the change in model output was compared to that produced by swapping random image patches of the same size. Neither replaced token produced a change in model output that was significantly greater than that expected from swapping random patches (top, empirical $p$-value $= 0.251$ based on Monte Carlo substitution of random image patches, $n$=1000; bottom, $p = 0.900$, $n$=1000) (see Methods Section 2.5).
